# The Evaluation of Genetic Diagnosis on High-Risk Fetal CAKUT

**DOI:** 10.3389/fgene.2022.869525

**Published:** 2022-05-31

**Authors:** Wanlu Liu, Xinwei Shi, Yuqi Li, Fuyuan Qiao, Suhua Chen, Ling Feng, Wanjiang Zeng, Dongrui Deng, Yuanyuan Wu

**Affiliations:** Department of Obstetrics and Gynecology, Tongji Hospital, Tongji Medical College, Huazhong University of Science and Technology, Wuhan, China

**Keywords:** CAKUT, whole-exome sequencing, prenatal diagnosis, (copy number variants), karyotype analysis

## Abstract

**Background:** It is challenging to make an accurate prenatal diagnosis for congenital anomalies of the kidney and urinary tract (CAKUT) because of its pathologic diversity. This study aims to evaluate the performance of whole-exome sequencing (WES) combined with karyotype analysis and copy number variations (CNVs) in diagnosing high-risk fetal CAKUT.

**Methods:** We conducted a retrospective study on prenatal diagnoses of CAKUT in our hospital from January 2020 to April 2021. The research studied 24 high-risk fetuses with CAKUT who were scanned by ultrasonography at the prenatal diagnosis center of Tongji Hospital affiliated to Tongji Medical College of Huazhong University of Science and Technology. The likely pathogenic gene variants were screened for the patients and their parents by multiple approaches, including karyotype analysis, CNVs and WES, and further verified with Sanger sequencing.

**Results:** ①We detected abnormal CNVs in 20.8% (5/24) of the fetuses but only 8.3% (2/24) fetuses had abnormal karyotypes. ②Of the 15 CAKUT fetuses, positive findings (40%) were detected by WES. Of the 9 high-risk fetuses with CAKUT (negative findings in ultrasound scan but with family history), we found abnormal variants (77.8%) through WES.

**Conclusion:** The application of CNVs and WES showed advance in prenatal diagnosis of CAKUT and the pathogenic gene variants were detectable especially for high-risk fetuses with negative ultrasound findings on CAKUT in the preliminary study. The applied strategy could be used to improve the accuracy of prenatal diagnosis for CAKUT in the future.

## Introduction

Congenital anomalies of the kidney and urinary tract (CAKUT) occurs in 3–6 per 1000 live births and can be syndromic or non-syndromic in both familial and sporadic cases (Livera et al., 1989; Hildebrandt, 2010). There is a broad spectrum of renal defects in CAKUT, which could be shown with diverse phenotypes such as hydronephrosis, renal agenesis, dysplastic kidney, duplex kidney, ectopic kidney, fused kidney, and ureteropelvic junction obstruction (Schedl, 2007). CAKUT can cause recurrent urinary tract infections in childhood, whereas CAKUT in adults may present with hypertension and/or proteinuria that can lead to progressive chronic kidney disease (Pennesi et al., 2020). Therefore, it is not just challenging to determine CAKUT in clinical diagnosis due to the diversity of CAKUT phenotypes, but also in the prenatal diagnosis.

The majority of CAKUT cases are asymptomatic and usually found in prenatal diagnosis by ultrasound scanning, while other cases were incidentally found in routine examination for other malformations (Al-Hamed et al., 2021). However, the accuracy of prenatal diagnosis for CAKUT was affected by many factors, such as the availability of familial history information, and the difficulty in determining typical symptoms for fetus *in utero*. With the development of next-generation sequencing technology, high-throughput sequencing has been considered as an alternative method for diagnosing complex diseases. For instance, whole-exome sequencing (WES), is an advantageous approach to identify De novo and compound heterozygous variants (Chandler et al., 2018; Fu et al., 2018). Suggested by the American College of Medical Genetics and Genomics (ACMG), next-generation sequencing has shown a higher sensitivity of diagnosis when comparing with the tradition gene testing, including karyotype analysis and copy number variation examination (Lei et al., 2020).

In this study, the combination of gene sequencing test and sonographic screening was conducted on high-risk fetuses with CAKUT in the first or second trimester of gestation. By analyzing the gene sequencings and clinical symptoms of 24 cases, our study demonstrated likely associated gene variations in the prenatal diagnosis of 24 cases and the outcomes could provide scientific supports for genetic diagnosis consultation and clinical intervention of CAKUT in the future.

## Methods

### Participant Recruitment and Editorial Policies

Our study was approved by the Research Ethics Committee of Tongji Hospital affiliated to Tongji Medical College of Huazhong University of Science and Technology. Legal consents were obtained from all the participants in the study.

### Patients Information and Ultrasound Scanning

The parents with/without high-risk factors leading to fetal CAKUT were recruited for the study between January 2020 and April 2021since their fetuses were found to have CAKUT with/without other structural anomalies in an initial prenatal ultrasound scan (at 16–35 weeks of pregnancy), and were confirmed by a second ultrasound scan in the prenatal diagnosis center (one of prenatal diagnosis referral centers in China) of Tongji Hospital affiliated to Tongji Medical College of Huazhong University of Science and Technology. Those with confirmed diagnoses (24 cases) were recruited in the study.

### Sample Collection

Out of 24 cases, amniotic fluid and blood samples were obtained in 22 cases from umbilical cord by amniocentesis and cordocentesis respectively during the ultrasound examination at second trimester of gestation. For the rest 2 cases from which the blood or amniotic fluid samples could not be collected, two pieces of fetal muscle tissue (2 × 2 cm, containing the skin) or umbilical cord (about 3 cm) were sampled after the termination of pregnancy. In addition, the blood samples of parents were obtained during the prenatal diagnostic procedure for all the studied cases.

### Extraction of Genomic DNA

Fetal genomic DNA was extracted from amniotic fluid samples or the tissues samples of fetuses using a DNA Extraction Kit (TianGen, Beijing, China) according to the manufacturer’s instructions and stored at −20°C for further analysis. The genomic DNA of the couples in all cases was extracted from peripheral blood sample using the same protocol/Kit. A Qubit Fluorometry (Thermo Fisher Scientific) and electrophoresis were applied to evaluate the quality and quantity of genomic DNA after DNA extraction.

### Fetal Karyotype Analysis and Copy Number Variations

Amniotic fluid and umbilical cord blood samples were used for chromosome G band karyotype analysis and CNVs test. Brieflfly, 50 ng of amniocyte DNA was fragmented and DNA libraries constructed by end fifilling, adapter ligation, and PCR amplifification. DNA libraries were subjected to massively parallel sequencing on the NextSeq 500 platform (Illumina, San Diego, CA) to generate approximately 5 million raw sequencing reads with genomic DNA sequences of 36 base pair in length. Using the hg19 genomic sequence as reference, a total of 2.8–3.2 million reads were uniquely and precisely mapped using the BurrowseWheeler algorithm. Mapped reads were allocated progressively to 20 kb bin sizes from the p to q arms of the 24 chromosomes. Counts in each bin were then compared between all test samples run in the same flow cell to evaluate copy number changes using previously described algorithms. Chromosome profiles were finally plotted as copy number (*Y*-axis) *vs.* 20- kb count windows (*X*-axis). Identified and mapped CNVs were interrogated against publicly available databases, including Decipher, Database of Genomic Variants (DGV), 1000 genomes, and Online Mendelian Inheritance in Man (OMIM), and their pathogenicity assessed according to the guidelines outlined by the American College of Medical Genetics (ACMG) for interpretation of sequence variants. Variants were classifified as either pathogenic, likely pathogenic, variants of uncertain signifificance (VUS), likely benign, or benign.

### Whole-Exome Sequencing and Familial Validation

The WES was performed for all the DNA samples. The sequencing data was used for analyzing likely pathogenic gene variation by the Polyphen and SIFT software. The genetic variants were further examined for parents by Sanger sequencing when the pathogenic gene variations were detected in the fetal samples.

## Results

### Demographic Characteristics

In total, 24 high-risk fetuses with CAKUT were investigated. Of these, 4 (16.7%) had isolated CAKUT; 11 (45.8%) had CAKUT associated with other structural anomalies and 9 (37.5%) had no significant anomaly in ultrasound scanning. Varied symptoms were demonstrated among the 15 CAKUT fetuses, including unilateral multipolycystic kidney dysplasia (5 cases), unilateral renal agenesis (4 cases), bilateral multicystic kidney dysplastic and unilateral hydronephrosis (2 cases), unilateral renal dysplasia (1 case), renal vascular malformation (1 case), adrenal malformation (1 case) and bilateral ectopic kidney (1 case) (Table 1 and Table 2).

**TABLE 1:** Clinical data of the CAKUT cases with positive (abnormal) ultrasound results.

No.	Age	History	Gestation weeks	G/P	NT (mm)	Fetuses with Urinary Malformation	Other defects
1	31	The couple had once induced labor because of fetal bilateral renal dysplasia	28 + 4	G3P1	0.3	Unilateral multicystic kidney dysplastic	Right ventricular intense spot
2	42	The couple had once induced labor because of fetal bilateral ectopic kidney	24 + 4	G3P1	1.6	Bilateral pelvic ectopic kidney	None
3	32	The mother was diagnosed as a carrier of thalassemia	23 + 1	G1P0	1.3	Unilateral multicystic kidney dysplastic	Left ventricular intense spot
4	32	None	24 + 1	G3P1	1.5	Unilateral renal agenesis	Single umbilical artery
5	31	The couple had once induced labor because of the fetus with trisomy 16	18 + 4	G3P0	0.7	Unilateral enlarged and echogenic kidney, renal dysplasia	Omphalocele, facial dysplasia
6	35	None	19 + 4	G2P1	4.6	Unilateral renal agenesis	Single umbilical artery, bilateral nasal bone loss, bilateral foot varus, left diaphragmatic hernia
7	33	The mother was diagnosed with congenital polycystic kidney	30	G2P1	1.2	Bilateral multicystic kidney dysplastic	Left hydronephrosis
8	38	None	32 + 5	G4P1	1.2	Bilateral multicystic kidney dysplastic	Left hydronephrosis
9	27	None	28 + 2	G2P0	1.3	Left renal accessory renal artery	Right ventricular intense spot
10	32	None	29 + 6	G2P0	1.3	Left adrenal gland space occupying lesions	None
11	26	None	21 + 3	G2P0	1.8	Unilateral multicystic kidney dysplastic	None
12	27	None	25 + 3	G1P0	1.8	Unilateral multicystic kidney dysplastic	None
13	34	None	26	G3P1	1.5	Unilateral multicystic kidney dysplastic	Oligohydramnios
14	23	None	23 + 6	G3P0	1.2	Unilateral renal agenesis	Right renal double arteries
15	23	None	18 + 1	G1P0	2.7	Unilateral renal agenesis	Single umbilical artery

**TABLE 2:** The clinical data of the cases that carried high-risk fetal CAKUT with negative (normal) ultrasound results.

No.	Age	History	Gestation weeks	G/P	NT (mm)	Fetuses with Urinary Malformation	Other defects
16	28	The couple had a boy with Alport syndrome who died on 5 days after birth	18 + 5	G4P2	1.6	None	None
17	37	The couple had a girl with congenital adrenal hyperplasia	18 + 5	G4P2	1.7	None	None
18	25	The couple had a boy with congenital glomerulonephritis	18 + 2	G3P1	2.1	None	None
19	29	The couple had a 10-years old girl with congenital nephrotic syndrome	17	G2P1	1.5	None	None
20	30	The mother was diagnosed with congenital spongiform kidney	38 + 1	G2P1	1.2	None	None
21	31	The father was diagnosed with clear cell renal carcinoma	23 + 1	G2P0	1.5	None	None
22	32	The couple had a 4-years old girl with congenital nephrotic syndrome	18 + 3	G2P1	1.1	None	None
23	39	The couple gave birth to a 12-years old boy with renal failure	21 + 3	G2P1	1.5	None	None
24	27	The father was diagnosed with von Hippel-Lindau syndrome	15 + 3	G1P0	1.0	None	None

### Abnormalities by Chromosome G Band Karyotype Analysis and Copy Number Variations

Out of 24 high-risk fetuses with CAKUT, karyotype analysis detected only 2 (8.3%) cases with chromosome number abnormality. However, abnormal CNVs were presented in 20.8% (5/24) of the fetuses. In the case1, we found a 1.38MB duplication on the chromosome12p13.33, determined as the variants of uncertain significance (VUS); In the case 2, a 0.42MB duplication in the chromosome 3p26.3 was detected and recognized as likely benign (LB). In the case 5 and 6, 95.67 and 78.08 MB duplication in the chromosome 13q12.11q34 and 18p11.32q23 were detected respectively, which was determined to be likely pathogenic according to the available reports. In the case 12, a 0.18MB duplication in the chromosome 18q22.3 was found and considered as VUS (Table 3 and Table 4).

**TABLE 3:** Identification of variants in 15 CAKUT cases.

NO.	Chromosome Karyotype	CNVs					WES					Outcome
		Type	Size (MB)	Classification	Gene	Mutation (Nucleotide)	Substitution (Amino Acid)	Zygosity	ACMG Classification	Origin of Inheritance	Type of Inheritance	
1	46, XN	Dup (Kumar et al., 2012) (p13.33)	1.38	VUS	—	—	N	—	—	—	—	Full-term birth
2	46, XN	Dup (Schedl, 2007) (p26.3)	0.42	LB	—	—	N	—	—	—	—	Termination
3	46, XN	—	N	—	*CPLANE1*	c.8746G > A	p. A2916 T	Het	Vus	Pat	AR	Full-term birth
						c.6695C > T	p. S2232F			Mat		
4	46, XN	—	N	—	*PHGDH*	c.743C > T	p. A248V	Het	Vus	Mat	AR	Full-term birth
5	47, XN, +13	Dup(13) (q12.11q34)	95.67	P	—	—	*_*	—	—	—	—	Termination
6	47, XN, +18	Dup(18) (p11.32q23)	78.08	P	—	—	*_*	—	—	—	—	Termination
7	46, XN	—	N	—	*PKD1*	c.2853+2T > C	_	Het	LP	Mat	AD	Termination
8	46, XN	—	N	—	*PKD1*	c.5824C > T	p. R1942C	Het	VUS	Mat	AD	Termination
9	46, XN	—	N	—	—	—	N	—	—	—	—	Full-term birth
10	46, XN	—	N	—	—	—	N	—	—	—	—	Full-term birth
11	46, XN	—	N	—	—	—	N	—	—	—	—	Full-term birth
12	46, XN	Dup (18) (q22.3)	0.18	VUS	—	—	N	—	—	—	—	Termination
13	46, XN	—	N	—	—	—	N	—	—	—	—	Termination
14	46, XN	—	N	—	—	—	N	—	—	—	—	Full-term birth
15	46, XN	—	N	—	—	—	N	—	—	—	—	Termination

VUS, variants of uncertain significance; LB, likely benign; P, pathogenic; LP, likely pathogenic; N,negative founding; Het, heterozygosity; Hom, homozygosity; WT, wild type; Pat, paternity; Mat, maternity; AD, autosomal dominant inheritance; AR, Autosomal recessive inheritance; XLR, X-linked recessive inheritance.

**TABLE 4:** Identification of Variants in 9 cases with high-risk fetal CAKUT.

No.	Chromosome Karyotype	CNVs				WES				Outcome
			Gene	Mutation (Nucleotide)	Substitution (Amino Acid)	Zygosity	ACMG Classification	Origin of Inheritance	Type of Inheritance	
16	N	N	*COL4A5*	c.1769A > C	p. K590 T	Het	P	Mat	XLR	Termination
17	N	N	*CYP21A2*	c.518T > A	p. I 173N	Het	P	Mat/Pat	AR	Full-term birth
18	N	N	*NPHSC2*	c. 647C > T	p. A216V	Het	P	Pat	AR	Full-term birth
19	N	N	*TRPC6*	c.242A > G	p. N81S	WT	VUS	Mat	AD	Full-term birth
20	N	N	—	—	—	N	—	—	—	Full-term birth
21	N	N	—	—	—	N	—	—	—	Full-term birth
22	N	N	*NPHS1*	c.3478C > T	p.R1160*	Het	P	Pat	AR	Full-term birth
23	N	N	*PAX2*	c.76dup	p. V26Gfs*28	WT	P	De-novo	AD	Full-term birth
24	N	N	*VHL*	c.280G > T	p.E94*	Het	P	Pat	AD	Termination

### Pathogenic Variations Detected by Whole-Exome Sequencing

Of the 15 CAKUT fetuses with observed anomalies, 1 case (6.7%) was identified with carrying a likely pathogenic variant in gene *PKD1*; 2 cases (13.3%) were detected with the presence of lethal gene variants and fetal chromosomes were trisomy 13 and trisomy 18, respectively; 3 cases (20%) were found with variants in gene *CPLANE1, PHGDH* and *PKD1*, known as VUS; The rest 9 cases (60%) showed negative in WES (Table 3).

Of the 9 fetuses with negative CAKUT-like phenotype but with family history, 6 cases (66.7%) were found to carry pathogenic variants; 1 case (11.1%) was detected with a variant in gene *TRPC6*, known as VUS; the other 2 cases (22.2%) did not show any notable gene variation by WES (Table 4).

### Sanger Sequencing and Pregnancy Outcome

All of the positive cases from WES were further confirmed with Sanger sequencing (Tables 3, 4). The results demonstrated different features of the detected gene variants possibly associated with CAKUT in some specific cases.

**FIGURE 1:**
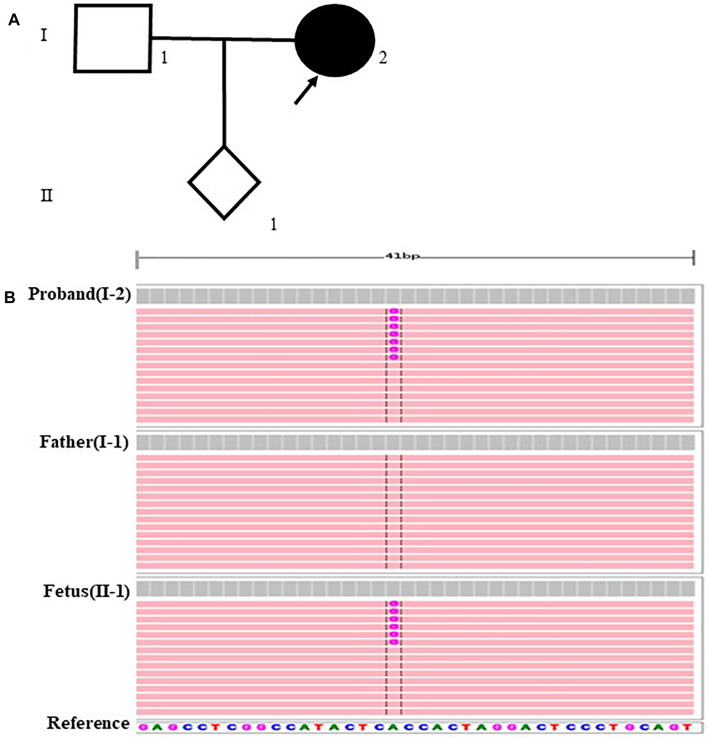
case7: **(A)** Pedigree of a family with Polycystic kidney disease 1. The empty symbols indicate unaffected individuals, and the filled symbols indicate affected individuals. The black arrow points out the proband (I-2). **(B)** Sequencing results showed that the proband and the fetus carried the splicing c.2853+2T>C in PKDI gene which was not present in the father.

The fetus of case 7 carried the splicing variant c.2853+2 T > C in *PKD1* gene indicated as likely pathogenic mutation to cause congenital polycystic kidney (Kim et al., 2021) and Sanger sequencing result showed that the variant was inherited from the mother but not the father (Figure 1). The parents decided to suspend their pregnancy after genetic counseling.

In the case 16, the couple had a son with Alport syndrome who died on the 5th day after birth. We found that their son (proband) carried the pathogenic mutation c.1769A > C (p. K590 T) and Sanger sequencing results showed that this variant inherited from the mother. During the second pregnancy, this pathogenic mutation was also detected in the fetus, who was identified as male. The parents decided to suspend their second pregnancy after genetic counseling since Alport syndrome was X-linked recessive inheritance (XLR). Later, they experienced the third pregnancy. The fetus detected with no suspected gene variation was born at full term and healthy in the follow-up survey for 1 year.

In the case 18, the couple had a son with congenital glomerulonephritis, who was detected pathogenic mutation c. 647C > T (p. A216V) and was a homozygote. During the second pregnancy, this pathogenic mutation was carried in the fetus, who was a heterozygote. The pregnancy was continued and completed after genetic counseling because the inheritance type of congenital glomerulonephritis was autosomal recessive inheritance. The newborn was healthy in the follow-up survey for 1 year.

**FIGURE 2:**
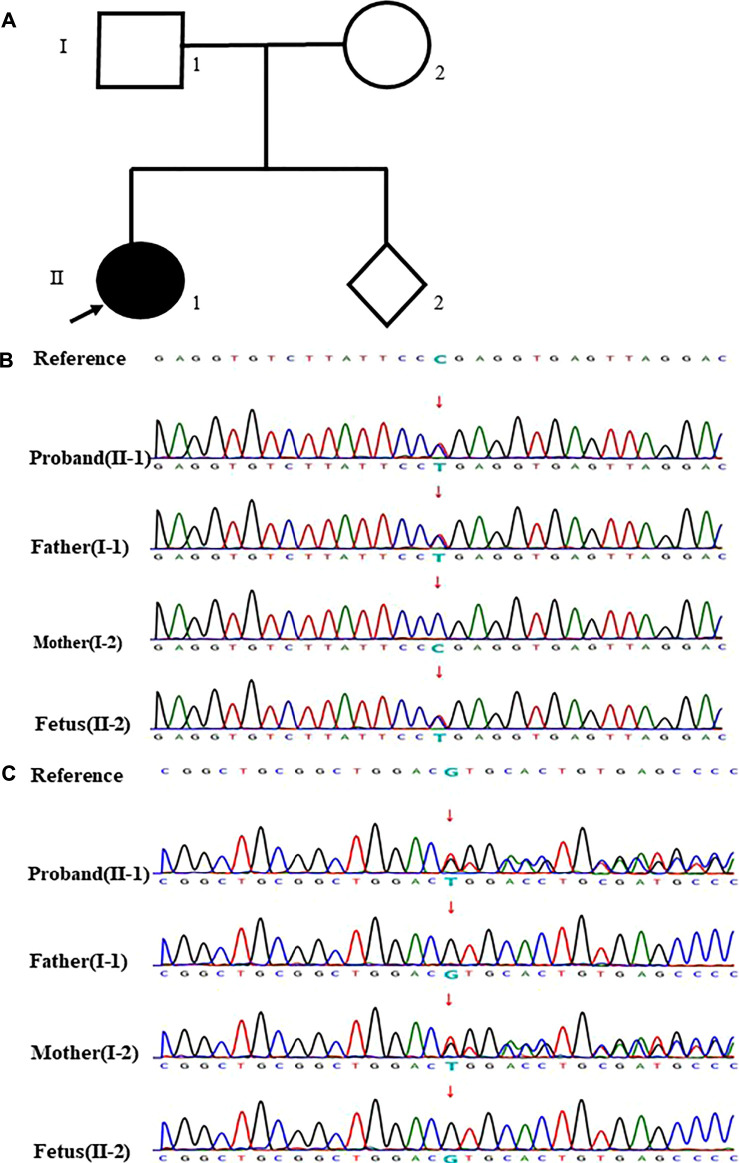
case22: **(A)** Pedigree of a family with congenital nephrotic syndrome. The empty symbols indicate unaffected individuals, and the filled symbols indicate affected individuals. The black arrow points out the proband (II-1). **(B)** Sequencing results showed that the proband, her father and the fetus carried the stopgain mutation c.3478C>T (p.R1160*) in NPHSI gene except her mother (the arrow showed the mutation site). **(C)** Sequencing results showed that the proband and her mother carried the frameshift insertion mutation ¢.2201_2205dup (p. V736Wfs*18) in NPHSI gene which was not present in her father and the fetus (the arrow showed the mutation site).

In the case 22, we found the fetus only carried the stopgain mutation c.3478C > T (p.R1160*) in *NPHS1* gene, which is known to be pathogenic mutation. Sanger sequencing further showed that this variant inherited from the father. In this family, we found the proband (II-1) not only carried the stopgain mutation c.3478C > T (p.R1160*) but also carried the frameshift insertion mutation c.2201_2205dup (p. V736Wfs*18) in *NPHS1* gene, which were inherited from the father and mother separately. In addition, the proband was diagnosed as congenital nephrotic syndrome but the clinical phenotype of parents was normal. Ultimately, the mother completed the pregnancy and gave birth at full term (Figure 2).

**FIGURE 3:**
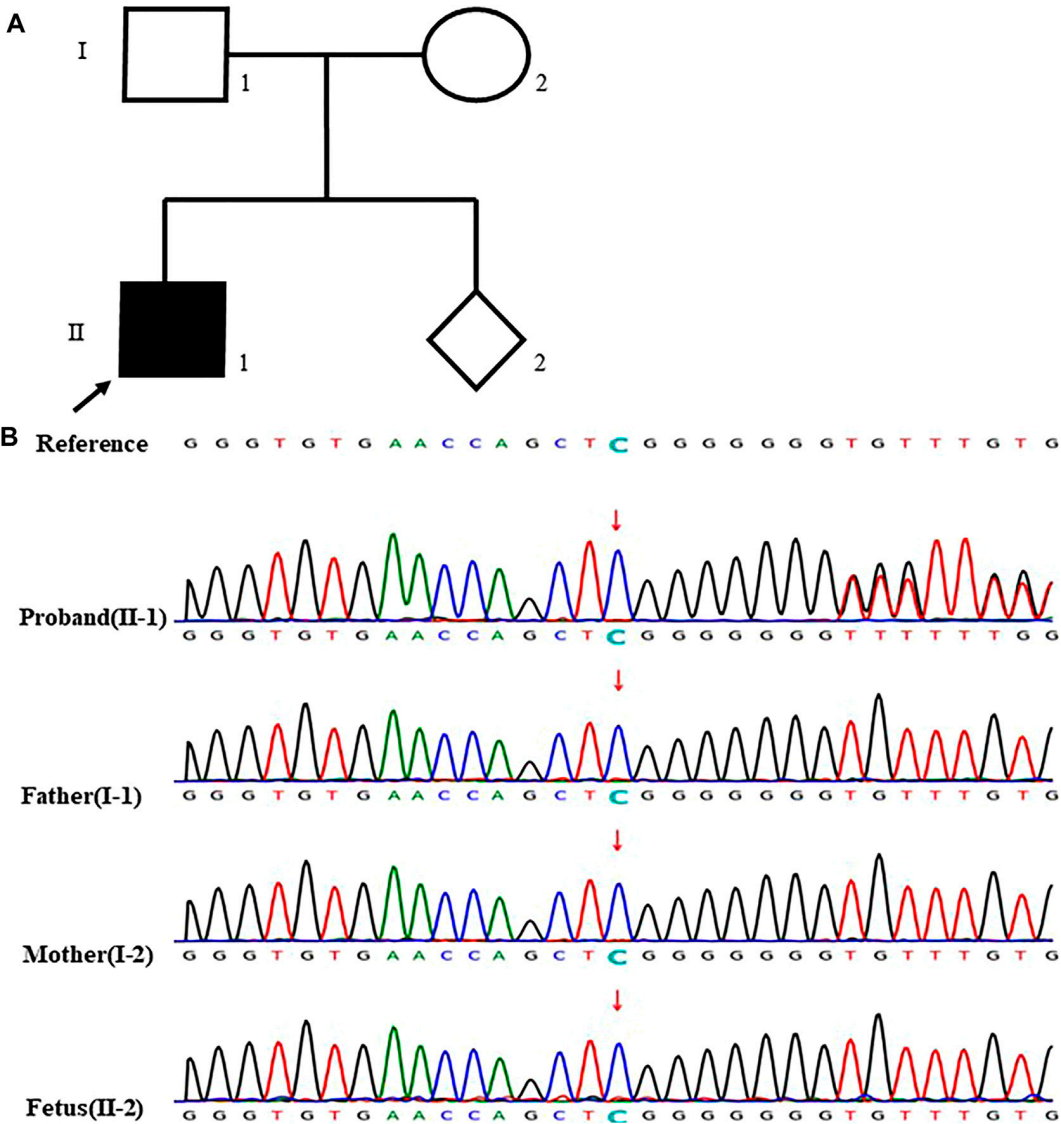
case 23: **(A)** Pedigree of a family with renal failure. The proband was diagnosed with renal failure at 12-years old. The empty symbols indicate unaffected individuals, and the filled symbols indicate affected individuals. The black arrow points out the proband (II-1). **(B)** Sequencing results showed that the proband carried the frameshift insertion mutation c.76dup (p. V26Gfs*28) in P4X2 gene which was not found in his parents and the fetus (the arrow showed the mutation site).

In the case 23, the analysis showed that the proband (II-1) with renal failure carried the frameshift insertion mutation c.76dup (p. V26Gfs*28) in *PAX2* gene, which was considered pathogenic mutation and De-novo mutation. However, the variant is not present in his parents and the fetus in the second pregnancy, indicating that the zygosity of fetus was wild-type. The parents decided to continue the pregnancy and a full-term baby was born (Figure 3).

**FIGURE 4:**
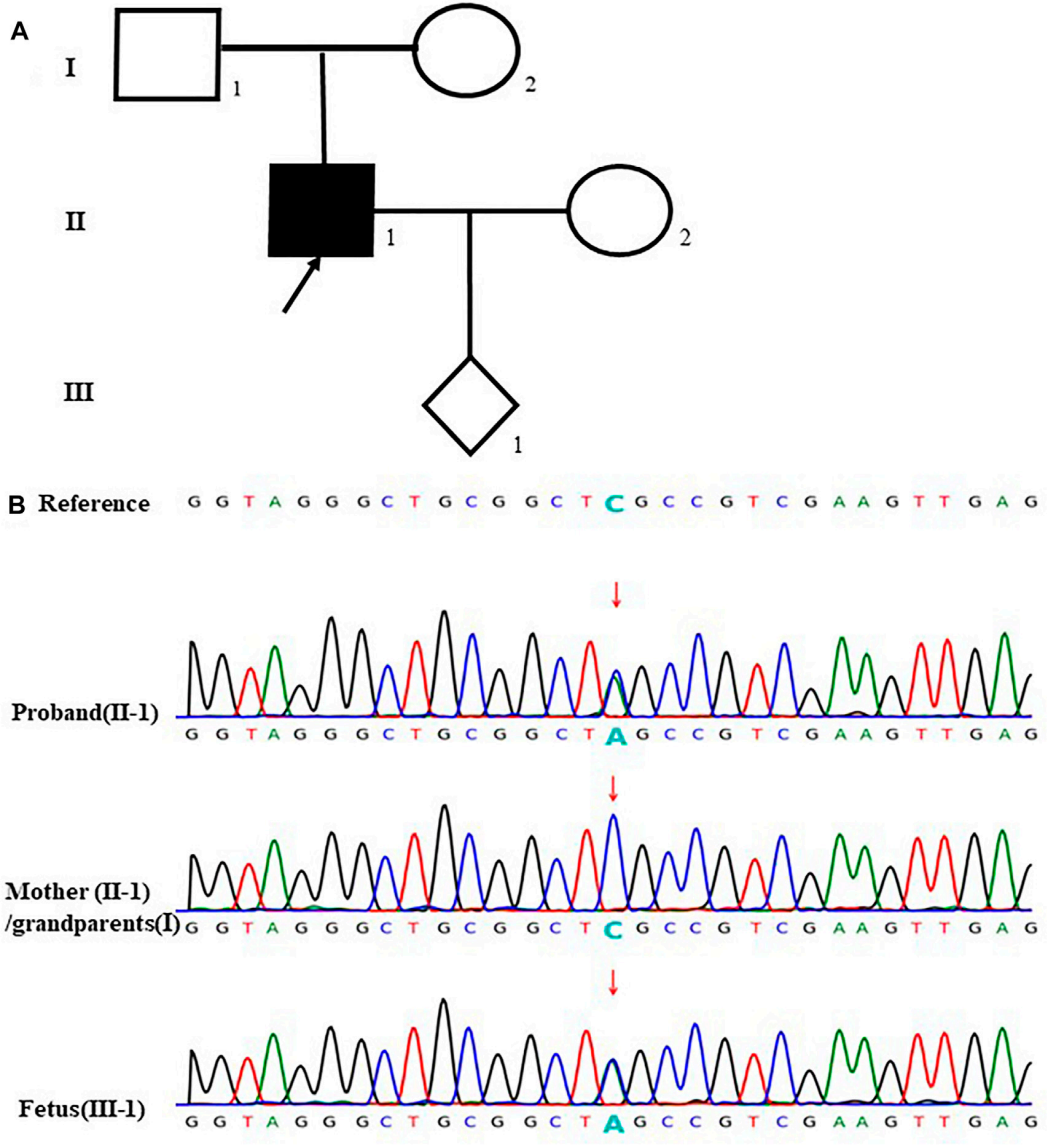
case24: **(A)** Pedigree of a family with von Hippel- Lindau syndrome. The empty symbols indicate unaffected individuals, and the filled symbols indicate affected out individuals. The black arrow points the proband (II-1). **(B)**Sequencing results showed that the proband (II-1) and the fetus (III-1) carried the stopgain mutation c.280G>T (p.E94*) in VHL gene which was not found in the mother (1-2) and the grandparents (I-1, I-2) (the arrow showed the mutation site).

In the case 24, the fetus (III-1) carried a novel stopgain mutation c.280G > T (p.E94*) in *VHL* gene considered as a pathogenic mutation. The sequencing result showed that the mutation was inherited from the father (II-1), which is unseen in the mother (II-2) and the grandparents (I-1, I-2). Besides, the father (II-1) was also diagnosed with von Hippel-Lindau syndrome which is autosomal dominant inheritance. The parents terminated this pregnancy since the fetus would be highly likely to have the disease (Figure 4).

## Discussion

Prenatal ultrasound scanning can detect signs such as oligohydramnios, microphthalmia, syndactyly, enlarged echogenic lungs, echogenic kidneys, renal dysplasia and renal agenesis (Vijayaraghavan et al., 2005). But these anomalies were usually detected late in pregnancy (particularly in the third trimester). Time of an effective diagnosis depends upon severity of the abnormality; for example, the mean time of diagnosis of bilateral renal agenesis was about 24 weeks of gestation, whereas the mean time of diagnosis of hydronephrosis was around 30 weeks (Queisser-Luft et al., 1998). In prenatal ultrasound scan, the most common renal anomaly was found to be hydronephrosis (Kumar et al., 2012). The second common anomaly was renal cysts (either bilateral or unilateral), followed by renal agenesis (unilateral > bilateral) (Kumar et al., 2014). In our study, the time of diagnosis of renal anomalies vastly ranged from 18^+1^ weeks to 32^+5^ weeks of gestation, with a mean time at 25 weeks. In fact, just one third of renal anomalies could be detected by prenatal ultrasound according to an extensive survey covering more than 20,000 fetuses and newborns (Queisser-Luft et al., 1998). The ultrasound-dependent approach is not effective to accurately diagnose and differentiate the complex types of CAKUT.

Prenatal gene diagnosis such as karyotypes, CNVs and WES, is added as a new dimension to determine CAKUT at early stage of pregnancy, which has proven to be an efficient tool to identity the underlying cause of congenital renal anomalies on molecular level. The development of CNVs technology has facilitated the diagnostic of genetic diseases which couldn’t be diagnosed by karyotypes. A few of studies reported that CNVs can be used to diagnose an additional 12–15% of genetic diseases in congenital structural malformations and neurocognitive developmental disorders (Melanie and Louanne, 2010; Vacic et al., 2011; Elia et al., 2012). Caruana *et al.* (Caruana et al., 2015) reported that 10.1% of individuals with CAKUT carried abnormal CNVs. In this study, we found abnormal CNVs in 20.8% (5/24) of the fetuses while only 8.3% (2/24) fetuses showed abnormal karyotypes. The diagnosis rate by CNVs examination was higher in our cohort than those in Caruana’s study. The different detection rates of pathogenic CNVs could attribute to differences in sample group, sample size, and the scales of array probes. Although the association between CAKUT and CNVs has been reported in fetuses, few studies noticed negative CNVs findings in fetuses with CAKUT. However, we reported negative CNVs in 9 cases associated with CAKUT.

Besides of conventional karyotypes and CNVs, the 24 high-risk fetuses with CAKUT and their parents was investigated in WES testing and Sanger sequencing for examining the complex of genetic variants of CAKUT. For the 15 CAKUT fetuses, positive findings (40%) were detected in 6 cases by WES. For the 9 high-risk fetuses with CAKUT, we found abnormal variants (77.8%) in 7 cases through WES. The results suggested that WES has greatly improved the diagnostic rate of CAKUT that cannot be diagnosed by conventional karyotypes or CNVs, especially for high-risk fetuses with negative ultrasound and CNVs findings.

## Conclusion

This is an initial study that analyzed the clinical and genetic features of high-risk fetuses with CAKUT using ultrasound and multiple sequence-based gene tests (including karyotype analysis, CNVs and WES). CAKUT is mostly hereditary. In this investigation, the likely pathogenic gene variants were found in the *PKD1, CPLANE1, PHGDH, TRPC6, NPHS1*and *VHL* genes. The combination of CNVs and WES showed advance for prenatal diagnosis of CAKUT in the preliminary study and the applied strategy could be helpful of improving the accuracy of prenatal diagnosis for CAKUT in the future.

## Data Availability

The original contributions presented in the study are included in the article/Supplementary material, further inquiries can be directed to the corresponding author.

## Abbreviations

AD, autosomal dominant inheritance; AR, Autosomal recessive inheritance; CAKUT, congenital anomalies of the kidney and urinary tract; CNVs, copy number variations; Het, heterozygosity; Hom, homozygosity; LB, likely benign; LP, likely pathogenic; Mat, maternity; N, negative founding; P, pathogenic; Pat, paternity; VUS, variants of uncertain significance; WES, whole-exome sequencing; WT, wild type; XLR, X-linked recessive inheritance.
